# Myometrial cavernous hemangioma with pulmonary thromboembolism in a post-partum woman: a case report and review of the literature

**DOI:** 10.1186/1752-1947-6-397

**Published:** 2012-11-23

**Authors:** Tapan Bhavsar, John Wurzel, Nahum Duker

**Affiliations:** 1 Department of Pathology and Laboratory Medicine, Temple University Hospital, Philadelphia, PA 19140, USA; 2 Fels Institute for Cancer Research and Molecular Biology, Temple University School of Medicine, Philadelphia, PA 19140, USA

**Keywords:** Hemangioma, Cavernous, Myometrium, Thrombosis, Pulmonary emboli

## Abstract

**Introduction:**

Cavernous hemangiomas of the uterus are rare benign vascular lesions. Nine cases of diffuse cavernous hemangioma of the gravid uterus have been reported, most of which diffusely involved the myometrium. These vascular malformations are clinically significant, and may cause pronounced bleeding resulting in maternal or fetal demise. Thrombosis of cavernous hemangiomas of the uterus has been previously reported. We here report the first case in which a thrombosed cavernous hemangioma of the myometrium resulted in a fatal pulmonary embolism in a post-partum woman.

**Case presentation:**

A 25-year-old obese African-American woman who had one pregnancy and was delivered of twins by cesarean section was admitted 1 week after the successful delivery. The 12-day clinical course included ventilator-dependent respiratory failure, systemic hypertension, methicillin-resistant *Staphylococcus aureus* in the sputum, leukocytosis and asystole. A transabdominal ultrasound examination showed heterogeneous thickened and irregular products in the endometrial canal. The laboratory values were relevant for an increased prothrombin time, activated partial thromboplastin time, ferritin and a decrease in hemoglobin. The clinical cause of death was cited as acute respiratory distress syndrome. At autopsy, a 400g spongy, hemorrhagic uterus with multiple cystic spaces measuring approximately 0.5 × 0.4cm filled with thrombi within the myometrium was identified. Immunohistological examination with a CD31 stain for vascular endothelium associated antigen confirmed several endothelium-lined vessels, some of which contained thrombi. These histological features were consistent with cavernous hemangioma of the myometrium. A histological examination of the lungs revealed multiple fresh thromboemboli in small- and medium-sized pulmonary arteries in the right upper and lower lobes without organization, but with adjacent areas of fresh hemorrhagic infarction.

**Conclusion:**

This case underscores the importance of a high index of suspicion in a pregnant or post-partum woman presenting with respiratory symptoms. Thrombosis of the cavernous hemangiomas of the gravid or post-partum uterus is a rare entity. This case is of interest because it indicates that this condition can be fatally complicated by embolization of the thrombi in the cavernous myometrial hemangiomas. Although delivery by conservative methods, as well as cesarean section, is possible without resorting to hysterectomy, occasionally, the consequences could be fatal as in this case.

## Introduction

Both capillary and cavernous hemangiomas rarely occur in the uterus. A search of the current literature reveals reports of fewer than 50 cases [1]. Cavernous hemangioma of the gravid uterus has been identified nine times [2], with the first case reported at autopsy in 1897. Such hemangiomas can be found at all levels of the uterine wall including perimetrium, myometrium and endometrium, with most cases diffusely involving the myometrium. As with those at other locations, cavernous hemangiomas of the uterus may be congenital, associated with hereditary hemorrhagic telangiectasis, or secondarily acquired due to surgical intervention, trophoblastic disease, pelvic inflammatory disease, endometrial carcinoma, or diethylstilbestrol ingestion [3,4].

Such cavernous hemangiomas are associated with numerous obstetric and gynecological complications, ranging from intermenstrual spotting, menometrorrhagia and infertility, to maternal and/or fetal demise, resulting from pronounced bleeding of the gravid uterus [5-8]. Occasionally, they may present as life-threatening hemorrhage, requiring immediate surgical intervention including hysterectomy [5]. Definitive diagnosis depends on histological examination of the uterus. Thrombosis, with or without organization, of cavernous hemangiomas has been reported [9,10]. However, to the best of our knowledge, embolization of thrombi from such cavernous hemangiomas has not been reported. Here, we describe the first case of a diffuse cavernous hemangioma of the uterus resulting in a fatal pulmonary thromboembolization.

## Case presentation

A 25-year-old obese African-American woman who had one pregnancy and was delivered of twins by cesarean section was admitted 1 week after the successful delivery. She had collapsed, and was found unresponsive with bloody frothy fluid issuing from her mouth. Her social history included smoking tobacco and marijuana. The clinical differential diagnoses were eclampsia and post-partum cardiomyopathy. The 12-day clinical course included ventilator-dependent respiratory failure, systemic hypertension, methicillin-resistant *Staphylococcus aureus* in the sputum, leukocytosis and asystole. She was treated with gentamicin, ampicillin, clindamycin, Dilantin® (phenytoin sodium), Lovenox® (enoxaparin), Ativan® (lorazepam), hydralazine, and morphine. Acute respiratory distress syndrome (ARDS) was attributed to an infectious source, and multiple antibiotics were administered. The critical condition of the patient did not allow for a magnetic resonance imaging (MRI) study to locate sites of infection.

A transabdominal ultrasonography (USG) examination showed heterogeneous thickened and irregular products in the endometrial canal. The laboratory values were relevant for an increased prothrombin time, activated partial thromboplastin time, ferritin and a decrease in hemoglobin. The clinical cause of death was cited as ARDS.

**Figure 1:**
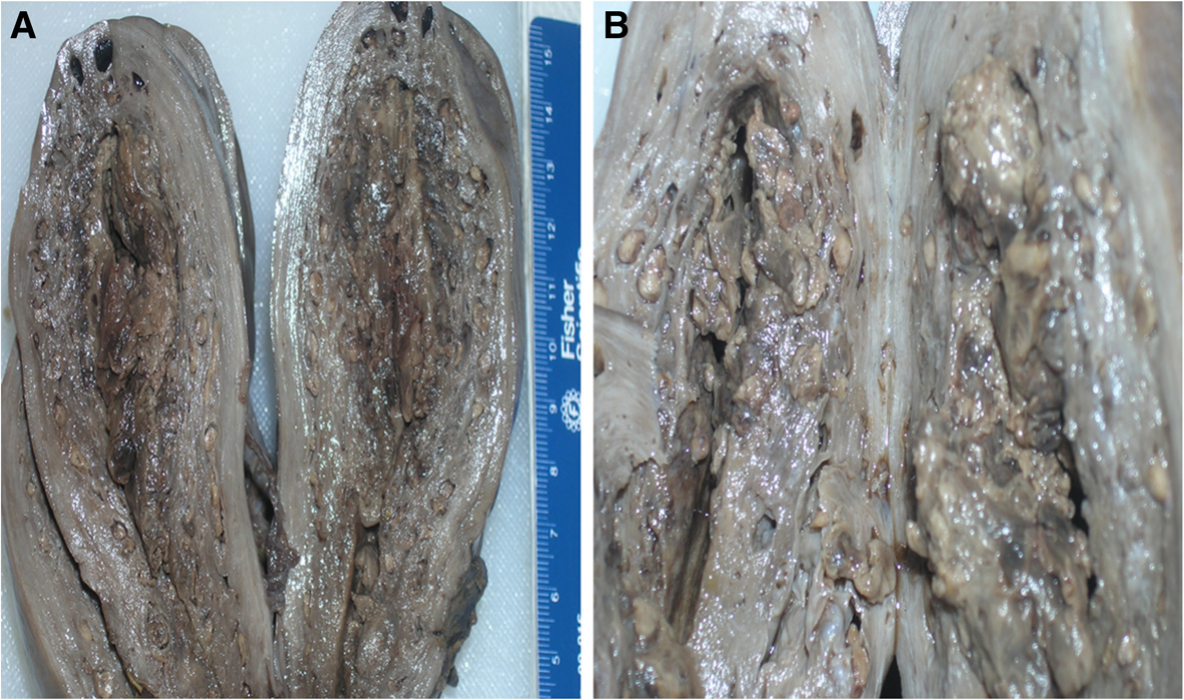
**(A, B): Uterus, gross, vertically sectioned in the sagittal plane.** Cut surface shows sponge-like appearance of diffuse network of dark blood spaces in the myometrium.

**Figure 2:**
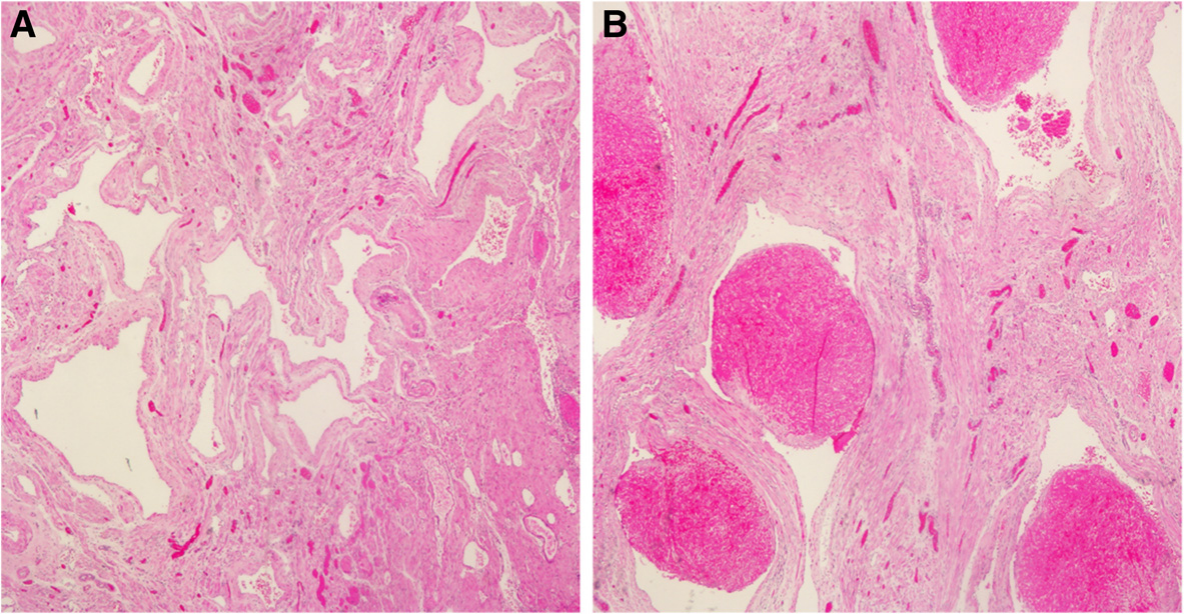
(A): Uterus, hematoxylin and eosin stain, original magnification 10×. (B) 40×.

**Figure 3:**
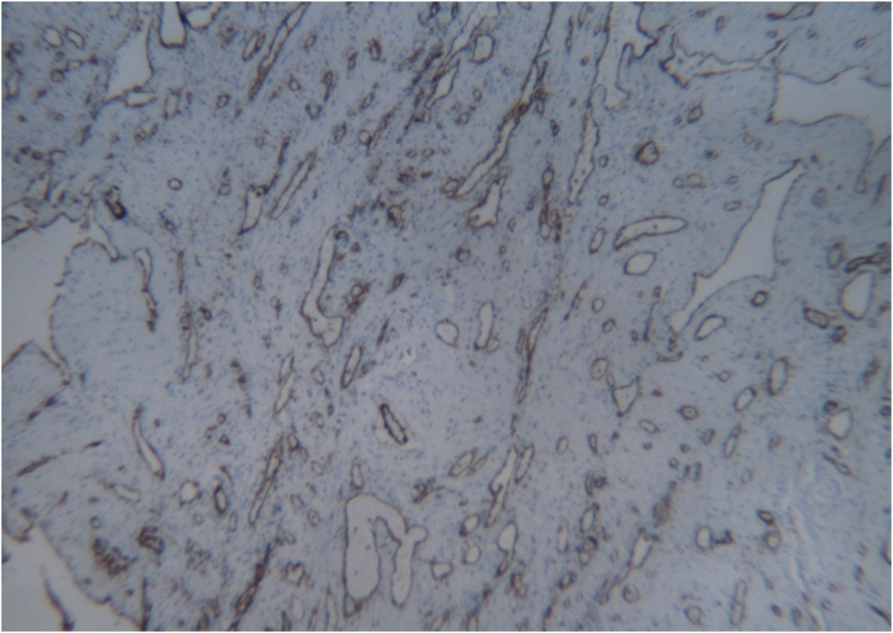
Uterus, immunohistochemistry, CD31, original magnification, 10×.

At autopsy, a 400g spongy, hemorrhagic uterus with multiple cystic spaces measuring approximately 0.5 × 0.4cm filled with thrombi (Figures 1, 2) within the myometrium was identified. Neither an abscess nor any retained placental tissues were present. Immunohistological examination with a CD31 stain for vascular endothelium associated antigen confirmed several endothelium-lined vessels (Figure 3), some of which contained thrombi. A Gram stain for bacteria and a Gomori methenamine silver stain for fungi were negative. Sections from the endometrium showed proliferative changes, hemorrhage and some endothelium-lined spaces. These histological features were consistent with cavernous hemangioma of the myometrium.

**Figure 4:**
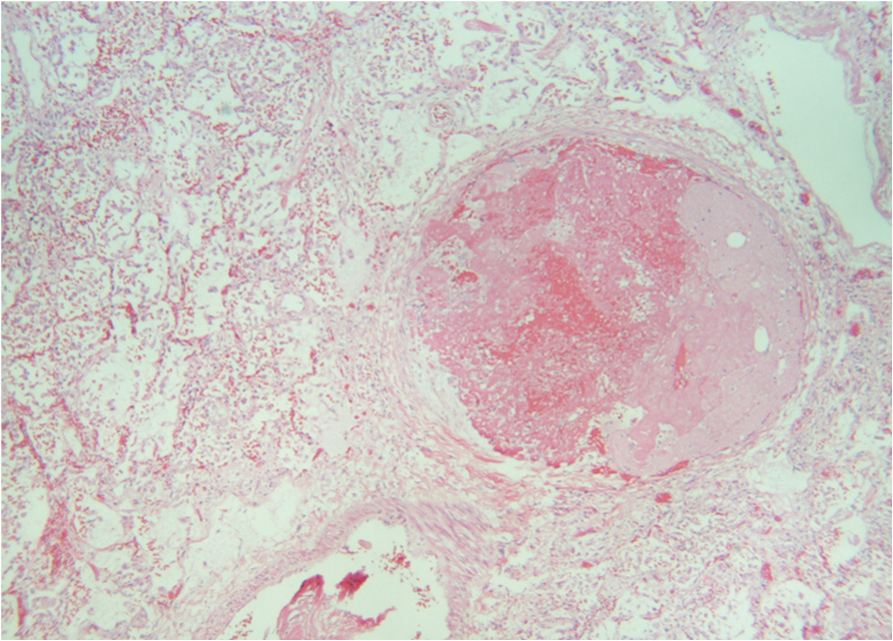
Lung, hematoxylin and eosin stain, original magnification, 40×.

Histological examination of the lungs revealed features of acute and early diffuse alveolar damage (DAD) and early organization with fibrosis. Multiple fresh thromboemboli in small- and medium-sized pulmonary arteries in the right upper and lower lobes without organization, but with adjacent areas of fresh hemorrhagic infarction, were identified (Figure 4). Immunohistochemistry for cytomegalovirus, herpes simplex virus-1 and herpes simplex virus-2 were negative; however, other viruses could not be ruled out. Neutrophils, granulomas and polarizable material were not identified. There were many interstitial and intra-alveolar macrophages identified by CD68 immunohistochemistry. There was no evidence of infection anywhere.

## Discussion

Hemangiomas are benign tumors [11] originating either from endothelial cells of blood vessels, or from pericytes located on the outer side of the vascular wall. They appear in two forms: capillary and cavernous. Capillary hemangiomas are usually on the skin; they vary in size and shape, and cause esthetic distortions. Histologically, they are characterized by a large number of anastomotic vascular spaces of irregular arrangements and sizes, lined with endothelial cells, with the lumina filled with blood or thrombi. Cavernous hemangiomas are found in skin, liver, kidney, breast, muscles, intestinal wall, brain and bones. Histologically, they are characterized by vascular spaces lined by endothelial cells. These are wider than the capillary types, occasionally taking the form of a cavern. Both types, capillary and cavernous hemangiomas, occur in the uterus. Capillary hemangiomas are usually restricted to the endometrium, whereas the cavernous form involves the uterus in a diffuse fashion. Complications such as thrombosis with organization and calcification may occur.

Hemangiomas of the uterus may occur at any age; the youngest patient reported in the literature was 14 years old [5]. Such hemangiomas may be diffuse or localized. The diffuse form usually involves all layers of the uterus, whereas a localized hemangioma can present as an endometrial polyp, or be restricted to the myometrium. It can rarely present as a cavernous hemangiomatous polyp [12].

Physiological changes related to pregnancy involve hypertrophy of the myometrium, but not hemangiomatosis [13]. During early pregnancy, cavernous hemangioma of the gravid uterus is a serious condition, and may not be detected. Clinically, these vascular lesions are usually asymptomatic, but might bleed spontaneously or following curettage; they can be associated with menstruation or termination of pregnancy. That the diagnosis is not usually considered is due to the rarity of this condition and vagueness of the symptoms. A few cases have been confused with disseminated intravascular coagulation (DIC) or uterine atony. DIC leads to the formation of fibrin thrombi in the microvasculature of the body, including the alveolar capillaries in the lungs. There were no microthrombi identified in our case; instead, the thromboemboli were evident in the small- to medium-sized pulmonary arteries. In a variation of the theme, in extremely rare cases, giant hemangiomas could be associated with an unusual form of DIC, in which thrombi form within the neoplasm. This is attributed to stasis and recurrent trauma to fragile blood vessels. It is difficult to intellectualize if such a rare phenomenon could have been contributory to the cavernous thrombosis in this case. A high index of suspicion is required both of radiologist and obstetrician in case of heavy bleeding per vaginam because delay in diagnosis can result in significant blood loss requiring a hysterectomy [1].

USG can reveal a thickened uterine wall and a mixed-echo texture suggestive of cavernous changes with turbulent flow [6]. Doppler, MRI and computed tomography imaging may be used in conjunction with USG for diagnosis. Arteriography has been successfully used in the diagnosis of unexplained cases of extensive refractory uterine bleeding in young patients. Therapeutic embolization of feeding vessels by liquid polymers helps preserve the reproductive capability with uncomplicated pregnancy [14].

Conservative treatments including carbon dioxide laser excision, knife excision, cryotherapy, radiotherapy, electrocauterization and uterine embolization have been attempted with varying results. In cases of uncontrolled bleeding especially during operative delivery, or not responding to conservative treatment, a hysterectomy is imperative. Successive pregnancies have been reported in women harboring cavernous hemangiomas of the uterus [15].

In cavernous hemangiomas of the uterus, the uterine wall is partly or completely transformed into cavern-like arteriovenous fistulas as a result of a proliferation of arterial and venous vessels of various sizes which later replace the normal myometrium [15]. A histopathological diagnosis of cavernous hemangiomas of the uterus usually occurs after a hysterectomy showing vascular spaces limited by endothelium assuming a cavern-like shape separated by connective septa.

Cavernous hemangiomas may be complicated by thrombosis, and such patients may develop DIC [9] due to platelet entrapment by abnormally proliferating endothelium within the hemangiomas. In pregnant women with congenital hemangiomas, hormonal alterations and physiological increases in blood volume may play contributory roles. Similar changes during pregnancy resulting in venous thrombosis in the myometrium have also been reported [10].

This is the first case of a thrombosed cavernous hemangioma of the uterus resulting in fatal pulmonary thromboemboli. Grossly, a 400g spongy and hemorrhagic uterus was identified. Histopathological examination revealed multiple vascular spaces within the myometrium lined by endothelial cells. Immunohistological examination with a CD31 stain for vascular endothelium associated antigen confirmed the endothelium-lined vessels, a few of which were filled with thrombi. The histologic features were in accordance with the previously reported cases of cavernous hemangioma of the uterus [1,2]. Histological study of the lumina revealed multiple fresh thromboemboli without organization in both small- and medium-sized pulmonary arteries in the right upper and lower lobes with adjacent areas of fresh hemorrhagic infarction. Features of acute and early DAD and early organization with fibrosis were also identified. The sources of the emboli were the extensive thrombosed cavernous myometrial hemangiomas, as no thrombi were identified in the popliteal or in other veins. The migration of these emboli through the channels of uterine, hypogastric and common iliac veins resulted in pulmonary embolism.

The proximate cause of death was attributed to terminal thromboemboli from cavernous hemangiomatosis of the myometrium and pulmonary infarcts with severe DAD. Although thrombosis of the cavernous hemangiomas has been previously reported [9,10], subsequent embolization was present in those cases.

## Conclusion

This case underscores the importance of a high index of suspicion in a pregnant or post-partum woman presenting with respiratory symptoms. Cavernous hemangioma of the gravid or post-partum uterus is a rare entity which conventionally can be complicated by refractory uterine bleeding and corresponding symptoms of acute blood loss. Thrombosis of the cavernous hemangiomas is not uncommon. This case is of interest because it indicates that this condition can be fatally complicated by embolization of the thrombi in the cavernous myometrial hemangiomas. These can be undiagnosed clinically, as happened here. Cavernous hemangiomatosis of the uterus is a very rare condition in a pregnant or post-partum woman. Although delivery by conservative methods, as well as cesarean section, is possible without resorting to hysterectomy, occasionally, the consequences could be fatal as in this case.

## Consent

Written informed consent was obtained from the deceased patient’s next of kin for publication of this case report and any accompanying images. Copies of the written consents are available for review by the journal’s Editor-in-Chief.

## Abbreviations

ARDS: Acute respiratory distress syndrome; DAD: Diffuse alveolar damage; DIC: Disseminated intravascular coagulation; USG: Ultrasonography.

## Competing interests

The authors declare that they have no competing interests.

## Authors’ contributions

TB conceived the case report, performed the gross examination of the specimen, acquired the patient’s data, searched the literature, and drafted the manuscript. JW helped in the histopathological evaluation of the slides, and made critical revisions to the manuscript. ND performed the histopathological evaluation of the slides, and made a critical analysis of the manuscript. All authors read and approved the final manuscript.
